# The Trini Sing-Song: Sociophonetic variation in Trinidadian English prosody and differences to other varieties^1^

**DOI:** 10.1177/0023830921998404

**Published:** 2021-03-19

**Authors:** Philipp Meer, Robert Fuchs

**Affiliations:** English Department, University of Münster, Germany;; Institute of Language Studies, University of Campinas, Brazil; Department of English, University of Hamburg, Germany

**Keywords:** Trinidadian English, prosody, pitch range, sociolinguistics, variation and change

## Abstract

The current study provides a phonetic perspective on the questions of whether a high degree of variability in pitch may be considered a characteristic, endonormative feature of Trinidadian English (TrinE) at the level of speech production and contribute to what is popularly described as ‘sing-song’ prosody. Based on read and spontaneous data from 111 speakers, we analyze pitch level, range, and dynamism in TrinE in comparison to Southern Standard British (BrE) and Educated Indian English (IndE) and investigate sociophonetic variation in TrinE prosody with a view to these global F0 parameters. Our findings suggest that a large pitch range could potentially be considered an endonormative feature of TrinE that distinguishes it from other varieties (BrE and IndE), at least in spontaneous speech. More importantly, however, it is shown that a high degree of pitch variation in terms of range and dynamism is not as much characteristic of TrinE as a whole as it is of female Trinidadian speakers. An important finding of this study is that pitch variation patterns are not homogenous in TrinE, but systematically sociolinguistically conditioned across gender, age, and ethnic groups, and rural and urban speakers. The findings thus reveal that there is a considerable degree of systematic local differentiation in TrinE prosody. On a more general level, the findings may be taken to indicate that endonormative tendencies and sociolinguistic differentiation in TrinE prosody are interlinked.

## 1 Introduction

The last two decades have seen a growing body of research concerned with prosodic variation across many of the varieties of English spoken around the world (e.g., Deterding, 2001; R. Fuchs, 2016; Gut, 2005; Low, Grabe, & Nolan, 2000). However, much of this research has focused on Inner Circle varieties (Kachru, 1985) such as British, American, Australian, and New Zealand English(es) (see Grice et al., 2019 for a comprehensive overview) and developed dedicated theoretical models and frameworks of intonational phonology based on these varieties (e.g., Crystal, 1969; Pierrehumbert, 1980). Comparatively less research exists on the prosodic systems of so-called New Englishes (Mufwene, 1994), that is, the postcolonial varieties of English spoken in different parts of Asia, Africa, the South Pacific, and the Caribbean, especially those with fewer numbers of speakers.

A central question in the study of these New Englishes is that of whether and to what degree individual postcolonial varieties develop, use, and publicly sanction their own, distinctive linguistic forms—sometimes called endonormativity. Evolutionary models of postcolonial Englishes, including Schneider’s (2007) influential Dynamic Model, generally assume that—in later stages of their evolution, typically (well) after the establishment of political independence—speakers of these varieties frequently rely on endocentric linguistic norms: at the level of language use, speakers increasingly adopt and produce local forms of English even in formal contexts, such that the emerging New English variety is “recognizably distinct” from the former (British) colonial standard in a number of respects (Schneider, 2007: 50–51). At the level of language attitudes, these local forms are more and more accepted and viewed positively (Schneider, 2007: 49). In parallel to these endonormative tendencies, New Englishes at this stage of development may be characterized by a certain degree of homogeneity (Schneider, 2007: 51), although this need not necessarily be the case (Van Rooy, 2014). A higher degree of local differentiation is then typically expected at a later developmental stage, after endonormative stabilization has been achieved (Schneider, 2007: 52–55). Very recent advances in World Englishes modeling (Buschfeld, 2020; Meer & Deuber, 2020; Schröder & Zähres, 2020), however, show that these norm stabilization processes can also be multidimensional in that they involve other global and local linguistic influences and co-occur with processes of differentiation.

In the English-official Caribbean, in line with the Dynamic Model, endonormative developments away from the former colonial British standard can generally be observed across several of the emerging standard varieties of English in the region (e.g., Deuber, 2013; Hackert, 2016: 105-106). However, these linguistic re-orientation processes tend to be context-specific and occur in an interplay of different local and non-local linguistic influences (e.g., Mair, 2002: 36, 2009; Meer & Deuber, 2020; Westphal, 2017), including: (remaining) British, and, in some domains, American English influences as well as influences from the region’s English-based Creoles, which coexist with local standard varieties of English and are linked to them by dialectal continua in most Caribbean islands (Deuber, 2014: 11).

Research in support of these observations comes from investigations of language attitudes and language use. Language attitude studies indicate that the idea of national endonormative standards in the Caribbean is generally gaining ground but that exonormative, especially British, influences still play a role in what is typically considered standard (e.g., Belgrave, 2008; Deuber, 2013; Deuber & Leung, 2013; Meer et al., 2019; Oenbring & Fielding, 2014, p. 45; Westphal, 2017; Wilson, 2017). At the same time, (extensive) Creole influence on standard English usage is usually deprecated (e.g., Deuber & Leung, 2013; Westphal, 2017; Wilson, 2017)—despite the (partial) rise in prestige of Caribbean Creoles in the post-independence period and the general easing of attitudes of what was formerly often regarded as merely “bad” or “broken” English (Beckford Wassink, 1999; Jamaican Language Unit, 2005; Oenbring & Fielding, 2014; Rickford & Traugott, 2019; Salmon & Gómez Menjívar, 2016; Winford, 1976). Studies of spoken language use have mostly been concerned with variation at the morphosyntactic (e.g., Deuber, 2014; Mair, 2009) and segmental phonological level (e.g., Ahlers & Meer, 2019; Irvine-Sobers, 2018; Kraus, 2017; Leung, 2013; Rosenfelder, 2009) and provided consistent evidence of distinct (often Creole-influenced) features and local innovations, especially at the phonological level, and an increasing reliance on these endocentric forms of English. Nevertheless, accents of standard English in the region are also often characterized by some degree of exonormative influence: while American and British influences have a prominent role in some domains, such as radio newscasting across different Caribbean islands (Deuber & Leung, 2013; Hänsel & Deuber, 2019; Hänsel, 2021; Westphal, 2017), these influences seem to be smaller on a more general level and standard British English mostly tends to be the somewhat stronger exonormative force (see Leung, 2013 on Trinidad; Rosenfelder, 2009 on Jamaica).^2^

Fewer studies have targeted speech prosody. In Trinidadian English (TrinE), however, variation in prosody may be an important dimension at which endonormative tendencies come to the fore. Early descriptive work, for instance, largely based on anecdotal evidence, has claimed that Trinidadian speech is characterized by a higher pitch level and wider range throughout the intonation phrase compared to what is commonly expected for English outside Trinidad (Winer, 1993: 20; see Wells, 1982: 573–575 for similar claims concerning varieties spoken in the Caribbean more generally). In particular, popular perceptions of TrinE prosody suggest that this may be a characteristic feature of the local variety of English. TrinE is frequently described as “sing-song” by non-linguists from Trinidad and abroad and considered to be distinct from other Englishes in this regard (Ferreira & Drayton, 2017: 24; Youssef & James, 2008: 334).

Following the long-standing focus on the region’s English-lexicon Creoles in linguistic research on the anglophone Caribbean, most studies on prosodic aspects of Caribbean varieties (e.g., Devonish, 2002; Gooden et al., 2009; Sutcliffe, 2003) have been concerned with Caribbean English Creoles. Similarly, in Trinidad, there is research into the prosodic system of Trinidadian English Creole (TEC) both from a synchronic and diachronic perspective (Drayton, 2013; Gooden & Drayton, 2017). By contrast, little research has investigated prosodic aspects of standard TrinE (or other Caribbean standard varieties). Those studies that exist have mostly been concerned with phonological descriptions of intonation (in TEC and other Creoles) and predominantly been based on limited numbers of speakers (e.g., Drayton, 2013; Sutcliffe, 2003; also see the description of TrinE prosody in Ferreira & Drayton, 2017: 19–25); analyses based on larger numbers of speakers and concerned with phonetic aspects of pitch modulation are rare. With its explicit focus on standard TrinE (as opposed to TEC) and phonetic aspects of variation in pitch, the present study aims to address this research void.

While TrinE is popularly described as “sing-song”, it is not fully clear which specific prosodic aspects contribute to this lay reaction to the variety. Both the phonological and the phonetic characteristics of TrinE and potential influences from TEC might play a role. On the phonological level, previous studies indicate that two intonational patterns might be partly responsible: first, TrinE is said to have a relatively high frequency of intonation phrase-final rises in declarative utterances (Ferreira & Drayton, 2017: 18; Youssef & James, 2008: 334). Second, an extensive study concerned with TEC prosody demonstrates that intonation phrases are commonly structured into accentual phrases marked by low (L*) pitch accents and high (H) boundary tones, resulting in frequent alternation between low and high tonal targets (Drayton, 2013). Drayton (2013: 260) argues that it is this accentual phrasing with recurring high and low tones that leads laypeople to describe TEC as “sing-song” or “lilting”. The prosody of (standard) TrinE is reported to be heavily influenced by that of TEC and show the same intonational pattern (Ferreira & Drayton, 2017; see also Youssef & James, 2008: 334), but this claim is still to be verified by large-scale empirical research on standard TrinE. At the same time, differences in the extent to which older and younger speakers make use of such patterns also seem to exist, at least in TEC (Gooden & Drayton, 2017; see Section 3).

On the phonetic level, the degree of variation in pitch might also contribute to TrinE “sing-song”. Variation in pitch in the local Trinidadian context seems to be perceptually salient and meaningful (Drayton 2013: 309; Leung & Deuber, 2014; Meer et al., 2019: 110–111; see Section 3 for details) and there is preliminary evidence that pitch in TrinE has a wider range than in British English (Wilson, 2007; see also observations reported in Winer, 1993: 20). There is also tentative evidence that pitch variation is sociolinguistically conditioned and varies along ethnic (and gender) lines in TrinE (Leung & Deuber, 2014). So far, no comprehensive analysis of sociolinguistic variation in TrinE prosody exists, and it is thus unclear whether a wide range in pitch is characteristic of TrinE as a whole or only specific speaker groups. Moreover, the degree of pitch variability (or dynamism) within the overall pitch range may play a role—as indicated by the finding that accentual phrasing with recurring alternation between low and high tonal targets seems to be shared between TrinE and TEC on the phonological level (Ferreira & Drayton, 2017). Phonetic information on pitch dynamism, however, is currently lacking (see also Drayton, 2013: 308–309 for calls to expand prosodic research on TrinE to phonetic aspects). In sum, therefore, there is currently inconclusive evidence on

(1) whether a wide pitch range and very dynamic pitch are characteristic, endonormative features of TrinE,
(2) to what extent TrinE differs from other varieties of English regarding these two aspects, and,
(3) how and to what extent pitch variation is generally sociolinguistically conditioned, in addition to previously observed differences along the lines of ethnicity and gender.

The present study will address these questions from the viewpoint of speech production by analyzing pitch level, range, and dynamism in standard TrinE (as opposed to TEC) in comparison to two other varieties of English—Standard Southern British English (BrE) and (Educated) Indian English (IndE)—and investigating sociophonetic variation across these global pitch parameters in TrinE. Given that standard speech in Trinidad (and other Caribbean territories) is usually found in more formal contexts only, with possible domain-specific differences (Deuber & Leung, 2013: 311, Meer & Deuber, 2020: 292–294, Westphal 2017: 221), the study relies on Trinidadian data collected from one of these domains: secondary school education, which is traditionally reserved for the standard variety and decisive for its inculcation (see Section 2 for details). The varieties of English investigated alongside TrinE in the present paper are important points of comparison. Although it is not the only external linguistic norm and its former influence as a colonial standard has certainly decreased, BrE still serves as a conservative norm for standard English usage in Trinidad (e.g., Deuber, 2013; Leung, 2013: 147–148; see above), and continues to carry high social status and prestige in the domain of education in particular (Meer et al. 2019). A historical form of BrE, moreover, is the superstrate variety of TrinE. By contrast, IndE has not directly influenced TrinE. However, TrinE has been influenced historically by substrate influence from Bhojpuri, the main substrate language historically spoken by Indo-Trinidadians (Mahabir, 1999: 14; Youssef & James, 2008: 323). While Bhojpuri is not among the main substrate languages that influenced the historical development of IndE, it is closely related to some languages that did (e.g., Hindi). In addition, regardless of the degree of genetic closeness or distance of Bhojpuri to other Indian languages, linguistic diversity on the Indian subcontinent is tightly constrained through continued contact and influence, a phenomenon known as the Indian sprachbund (Emeneau, 1974, 1980). In consequence, historical, contact-induced influence from Bhojpuri on TrinE—and on Trinidadian intonational phonology specifically (see Section 2)—may have striking similarities to that of Indian languages more generally on IndE. Similar to TrinE and in contrast to BrE, for instance, different sub-varieties of IndE mark pitch accents with low tones (Maxwell, 2014; Pickering & Wiltshire, 2000). The present paper thus provides a comparative phonetic perspective on pitch variation of TrinE with one variety whose intonational phonology is more similar (i.e., IndE) and one that is more different to that of TrinE, but historically influential (i.e., BrE). Additionally, while explicitly concerned with standard TrinE (and not TEC), the analysis of sociolinguistically conditioned prosodic variation in the current study is informed by previous findings on variation and change in the intonation of TEC (Drayton 2013; Gooden & Drayton 2017), considering that heavy influences of TEC on TrinE at the prosodic level are expected (Ferreira & Drayton 2017; also see Section 2). Specifically, drawing on comparable datasets for all three varieties, we address the following research questions:

RQ1 To what extent does TrinE differ from BrE and IndE in terms of global pitch parameters, namely pitch level, range, and dynamism, in both read and spontaneous speech?
RQ2 How homogenous is TrinE in terms of pitch variation? Which sociolinguistic factors influence variation across these global pitch parameters and to what degrees?

In the subsequent sections, we first provide information on the sociolinguistic profile of Trinidad (Section 2). Section 3 summarizes previous research on sociolinguistically conditioned prosodic variation in TrinE (and TEC), leading up to specific hypotheses (Section 4). Sections 5 and 6 describe our methods and results. In Sections 7 and 8, we discuss our findings with regard to our research questions and previous research and conclude with implications for the questions of endonormativity and the ‘sing-song’ quality of TrinE.

## 2 The Sociolinguistic Profile of Trinidad

Trinidad is the southernmost island of the Eastern Caribbean and, together with its considerably smaller sister island Tobago, forms the twin-island country of Trinidad and Tobago, which became independent from the United Kingdom in 1962. Trinidad has had a diverse (linguistic) history (Deuber, 2014: 28–29) with Spanish occupation until 1797, large numbers of francophone immigrants toward the end of that period, and subsequent British colonization and the arrival of English and English-lexicon Creole-speaking people from Britain and other Eastern Caribbean islands. At the end of the 19th and beginning of the 20th century, unlike other Caribbean islands, Trinidad experienced large waves of immigration of indentured laborers from India. The latter transformed Trinidad into an ethnically diverse island. According to the 2011 census, two ethnic groups make up the majority of Trinidad’s current population of around 1.27 million, namely Afro-Trinidadians (31.8 percent) and Indo-Trinidadians (37.0 percent), with another 7.8 percent having mixed Afro- and Indo-Trinidadian backgrounds (Republic of Trinidad and Tobago, 2012: 16).

The development of English and Creole in Trinidad thus occurred under the influence of many additional (adstrate) languages and language varieties, most notably, (Trinidadian) French Creole and (Trinidadian) Bhojpuri (Youssef & James, 2008: 322–323). The latter is said to have influenced considerably the intonational phonology of TEC (and TrinE), that is, initially Trinidadians of Indian descent and later on, by way of convergence between the ethnic groups, also Afro- and mixed-identifying Trinidadians (Gooden & Drayton, 2017; see Section 3). Today, languages other than TrinE/TEC are only spoken by a very small number of people in Trinidad, and Trinidad’s sociolinguistic situation is thus similar to that of many other Caribbean islands: standard English coexists with a (mesolectal) English-based Creole along a continuum of variation, while, on a functional level, both varieties have traditionally been in diglossic distribution (Winford, 1985), with TEC being associated with informal, oral and TrinE with formal domains, such as education, news media, business, and politics (Youssef, 2004: 44). However, similar to other Caribbean islands, this functional separation is increasingly transformed, such that Creole can now also be heard in a variety of different domains (e.g., Mühleisen, 2001).

As mentioned above, the present study relies on data obtained from one of these domains, namely the secondary school setting. School education in Trinidad generally follows what Craig (1980) termed “transitional bilingualism”: as standard English is usually only (fully) acquired in the education system, considering that many students have limited exposure to standard English outside the classroom, TEC is accepted as medium of expression for students in the early years of schooling (Youssef, 2014: 182). However, later in secondary school education there is an increasing and consistent demand for the standard. Although some Creole may also be spoken in secondary schools, including but less so by teachers (Deuber, 2009; Youssef, 2014), the underlying norm of the teachers and advanced secondary school students analyzed here is standard English (see Deuber, 2013:126; Republic of Trinidad and Tobago, 2015 on curricular requirements).

At the same time, in parallel to the influences of Creoles on varieties of English in other Caribbean islands (Hänsel, 2021, Irvine-Sobers, 2018; Kraus, 2017; Mair, 2002: 36; Rosenfelder, 2009; Westphal, 2017), standard TrinE has been shown to be influenced by TEC. Direct influences from TEC on standard TrinE are not only reported at the prosodic (Ferreira & Drayton, 2017; Youssef & James, 2008: 334) but also at the segmental phonological level—both with regard to variation in vowels (Leung. 2013) and consonants (Youssef & James, 2008: 329). Crucially, however, the degree of Creole influences in Trinidad and other Caribbean Creole continua settings is dependent on the specific domain of use and mediated by social and stylistic factors; in more formal contexts, in fact, fewer and more indirect Creole influences may often be observed (Deuber, 2014; Leung, 2013; Wilson, 2014; see Westphal, 2017 on Jamaican English). In these contexts, as previous research from speech production and perception has shown, standard English may be defined negatively by its distance from Creole—or rather, what speakers may believe to be characteristic Creole forms (Deuber & Leung, 2013; Irvine-Sobers, 2018). Consequently, in more formal speaking styles, speakers targeting standard TrinE or other Caribbean standard norms have been found to systematically avoid stereotyped Creole features in particular (Leung, 2013: 129; Meer, 2019; Wilson, 2014; see Kraus, 2017 and Westphal, 2017 on other Caribbean varieties).

## 3 Sociolinguistic Variation in Trinidadian English and Creole Prosody

While a comprehensive study on sociolinguistic variation in TrinE and TEC prosody is still lacking, evidence from former and more recent sociophonetic studies concerned with variation on the segmental level (Leung, 2013; Meer, 2019; Winford, 1972) indicates that ethnicity, age, gender, level of education, rurality, and social class are all relevant variables. Apart from anecdotal evidence and informal observations, more controlled types of evidence come from studies in speech production and folklinguistics, with converging evidence that Indo-Trinidadians differ from Afro-Trinidadians in terms of typical pitch height and range, while age and gender function as potentially mediating factors. Folklinguistic evidence is provided by Stell (2018), who finds that “high pitches” are particularly associated with central and southern varieties in Trinidad. These areas have traditionally had a larger proportion of Indo-Trinidadian residents, which, in turn, is in line with the popular stereotype in Trinidad that Indo-Trinidadian speakers have higher- and Afro-Trinidadians lower-pitched voices (see Leung & Deuber, 2014; Stell, 2018: 131, 134). This observation was substantiated by a small-scale acoustic analysis of the global distribution of fundamental frequency (F0) in 16 male and female Afro- and Indo-Trinidadian speakers (four speakers in each cell; Leung & Deuber 2014) indicating that, while pitch level in TrinE overall compares to common reference values for English, differences exist between the ethnic groups, though only in the case of female speakers. Female Indo-Trinidadian speakers were shown to have both a higher pitch level (210 Hz vs. 166 Hz; operationalized as mean F0 in Hz) and larger pitch range (351 Hz vs. 258 Hz; operationalized as the difference between maximum and minimum F0 in Hz), with both minimum (120 Hz vs. 100 Hz) and maximum F0 (471 Hz vs. 358 Hz) being higher in Indo-Trinidadian speech.

As indicated by research in speech perception, these differences also appear to be enregistered and relevant for the perception of speakers as belonging to different social groups. An ethnicity identification experiment involving natural and modified stimuli with increased (Afro-Trinidadian) and decreased (Indo-Trinidadian) pitch levels further showed that pitch level is used as a perceptual cue to determine the ethnicity of both male and female speakers (Leung & Deuber, 2014), mostly confirming the popular stereotype at the level of speech perception. A verbal-guise study with Trinidadian secondary school students (Meer et al., 2019) provides tentative support along these lines. Female Afro-Trinidadian (compared to female Indo-Trinidadian) speakers included in the study were found to be downgraded for social attractiveness (but not social status) traits by Indo- compared to Afro-Trinidadian informants.^3^ In the absence of larger differences between speakers at the segmental phonological level, Meer et al. (2019: 110-111) suggest that it is not unlikely that differences in pitch level have allowed informants—possibly guided by the popular stereotype—to categorize speakers according to their ethnicity and demonstrate this type of ingroup loyalty in the accent ratings (see Mühleisen, 2001: 56 on ethnic in- and outgroup differences in the evaluation of Trinidadian speech). Yet, as pitch variation and ethnic group identification were not further examined per se, this interpretation needs to be considered preliminary. In sum, however, there are indications that pitch variation in TrinE in production and perception is conditioned both by speaker gender and ethnicity.

However, as Leung and Deuber (2014: 23) critically point out, their findings on speech production are based on a small number of speakers and require empirical substantiation based on larger datasets. Additional potentially relevant sociolinguistic factors (such as speaker age; see below) were deliberately not considered. We argue that these factors should be included in a systematic analysis of sociolinguistically conditioned prosodic variation in TrinE. Further avenues for empirical substantiation we consider therefore include (1) operationalizing pitch range in a way that is more robust to outliers in the F0 track than maximal pitch range, (2) measuring this range on a scale that is closer to human perception of F0 at different frequency levels than Hz (i.e., semitones), and (3) investigating pitch variability or dynamism within the overall pitch range of a speaker.

Through an investigation of differences between age groups in TrinE, we also investigate whether there is evidence for ongoing linguistic change in TrinE prosody. Specifically, following the concept of change in apparent time, age-stratified variation in linguistic behavior often reflects ongoing change, with older speakers representing a more distant point in time and younger speakers a more recent point (Bailey et al., 1991; Labov, 2006: 190-240). For TrinE prosody, such evidence is lacking, but Gooden and Drayton (2017) provide a diachronic perspective on intonational phonological variation in Trinidadian (and Jamaican) Creole based on present-day data and recordings from the 1970s. As reports indicate a high degree of direct influence from TEC on TrinE prosody (Ferreira & Drayton, 2017; Youssef & James, 2008: 324), a similar age-stratified pattern as well as variation along the same sociolinguistic lines might be expected in TrinE as in TEC. A key finding in Gooden and Drayton (2017) is that accentual phrasing marked with L* pitch accents and H boundary tones is generally well established in present-day TEC and (mostly) independent of a speaker’s ethnic background and residence in urban versus rural areas. Older speakers, in contrast, born at the end of the 19th or beginning of the 20th century showed marked ethnic differences. While Afro-Trinidadian speakers generally showed less consistent accentual phrasing and a greater presence of other types of pitch accents in the 1970s dataset, Indo-Trinidadian speakers mostly followed the intonational pattern observed in present-day TEC. The authors note that the observed pattern is likely a result of crosslinguistic/substrate influence from Bhojpuri in Indo-Trinidadian speakers, and that, in present time, the tendency toward accentual phrasing with frequent alternation between low and high tonal targets “is now further along in the speech of younger speakers” and “very possibly a marker of a new ‘Trinidadian’ identity among younger Trinidadians” (Gooden & Drayton, 2017: 436). Overall, these observations lead the authors to conclude that TEC intonational phonology is undergoing change in the form of ethnic convergence between Afro-, Indo-, and mixed-identifying Trinidadians toward more consistent accentual phrasing. We will investigate and test the hypothesis that similar patterns of ongoing linguistic change and ethnic convergence exist in TrinE prosody.

## 4 Hypotheses

Based on previous research, we formulate the following hypotheses:

1) Cross-varietal comparison of pitch variation
  H1) TrinE has a generally similar pitch level to that of other varieties of English (Leung & Deuber, 2014).
  H2) TrinE has a wider pitch range than other varieties of English, as indicated by the popular stereotype, anecdotal evidence (Wells, 1982: 573–575; see also Winer, 1993: 20), and a preliminary previous investigation (Wilson, 2007).
  H3) TrinE has an overall more dynamic pitch than other varieties of English, possibly as a phonetic consequence of accentual phrasing and frequent alternation between low and high tonal targets at the phonological level (Drayton, 2013; Ferreira & Drayton, 2017; Gooden & Drayton, 2017).
2) Sociolinguistic variation in TrinE prosody
  H4) Pitch variation in TrinE is not fully homogenous and differences to other varieties of English (BrE and IndE) have to be considered in light of local sociolinguistic variation in prosody. Global pitch parameters vary across age, gender, and ethnicity (Gooden & Drayton, 2017; Leung & Deuber, 2014), and possibly other factors but this remains to be seen.
  H5) As regards the degree to which these variables influence prosodic variation, we have the following expectations based on previous research:
    a) Indo-Trinidadian speakers have a higher pitch level and a wider range than Afro-Trinidadian speakers (effect: ethnicity; Leung & Deuber, 2014), and possibly, although this remains to be seen, more pitch dynamism.
    b) These differences, however, are larger for female speakers and considerably smaller for male ones (effect: ethnicity × gender; Leung & Deuber, 2014).
    c) Given that, on a phonological level, younger speakers more consistently use accentual phrasing with corresponding frequent variation between low and high tonal targets in TEC (effect: age; Gooden & Drayton, 2017), we expect that, assuming that there are correlates on a phonetic level and strong influences from TEC on TrinE (Ferreira & Drayton, 2017), younger speakers have a wider range and more dynamic pitch than older speakers.
    d) Specifically, based on Gooden and Drayton’s (2017) observation of change in progress in the intonational phonology of TEC in the form of ethnic convergence (effect: age × ethnicity), we expect to observe a similar change in apparent time in TrinE: younger speakers across all ethnic groups have similarly dynamic pitch and a higher range, while ethnic differences between older speakers are larger.

## 5 Method

### 5.1 Data

Read and spontaneous data from 69 female and male speakers of TrinE taken from a corpus of sociolinguistic interviews with Trinidadian secondary school teachers and advanced students is analyzed. The data were collected by the first author during two field trips in 2015 and 2016. The selection of teachers and advanced students as speakers in the Trinidadian dataset was specifically motivated by the fact that education is one of the domains in Trinidad where standard TrinE (rather than TEC) is spoken (see Section 2). All speakers were recorded with Zoom H4n recorders via SHURE cardioid MX150B/C microphones placed near a speaker’s sternum. The recordings had a good signal-to-noise ratio with little background noise.

The data are compared to similar datasets from BrE (22 speakers) taken from the DyViS Project (Nolan et al., 2006) and the Cambridge component of the IViE corpus (Grabe, et al., & Nolan, 2001) and IndE (20 speakers) originally analyzed in R. Fuchs (2018). All datasets contain recordings of speakers reading out a text passage and producing spontaneous speech in comparable interview settings. In total, the present study thus draws on data from 111 female and male speakers (see Table 1 for an overview). While the stimuli used in the BrE DyViS data and the IndE data from R. Fuchs (2018) were identical, they differ from those used to elicit TrinE speech, which in turn differ from those used in the IViE corpus. Differences in the texts and prompts may have induced some variability between the datasets due to dissimilar frequencies of specific sentence types. For example, the IViE reading passage contained 14 questions (or 12% of all sentence-final punctuation), while the DyViS and TrinE reading passages contained no questions. The spontaneous data consisted of free dyadic conversations (IViE), a structured police interview involving map task elements (DyViS) and, in the case of the Trinidadian data, semi-structured, directed conversation involving meta- and sociolinguistic topics such as language use at home, with friends, or in the classroom.^4^ While these tasks are not identical, all of them prompted speakers to mainly produce declarative sentences.

**Table 1.** Number of speakers by gender and variety of English.

Variety	Gender		Total
	Male	Female	
British English	16	6	22
Indian English	10	10	20
Trinidadian English	24	45	69

The TrinE dataset contains recordings of speakers from secondary schools throughout the entire island (25 teachers and 44 advanced students), covering a range of age groups and all major ethnic groups, namely Afro-, Indo-, and mixed-identifying Trinidadians (see Table 2 for details). Most of the schools and speakers (*n* = 62) were located in the metropolitan region ranging from the capital, Port of Spain, to Arima in north Trinidad, the most populous area in the island (Republic of Trinidad and Tobago, 2012: 44). Two schools from the city of San Fernando in the south (one speaker each) and one school from the rural area on the northern coast (five speakers) were also included in the sample; speakers from central and eastern parts of Trinidad were not sampled. The schools also differed extensively in the socioeconomic background of their student body and in terms of school type, ranging from schools that are usually labeled “prestige schools” in Trinidad, that is, denominational board schools with high-performing students from mostly (upper) middle and upper-class backgrounds, over average governmental secondary schools to schools with a large proportion of students with lower socioeconomic backgrounds. Two-thirds of the speakers were affiliated with prestige (*n* = 46) and one-third with non-prestige schools (*n* = 23). Approximately one-third of the teachers (but none of the students) in the sample (*n* = 9; 13% of all speakers) had lived two or more years abroad in the United States, Canada, England, or another English-official Caribbean island—in many cases for the purpose of tertiary education. Given that a high degree of outward mobility and (re-)migration is common in the Trinidadian population, especially among highly educated Trinidadians such as teachers and other former tertiary-level students (United Nations, 2018; UNESCO, 2018), these speakers were deliberately retained in the corpus. Excluding these mobile speakers from further analysis would not adequately represent the entire range of speakers of standard English in Trinidad, at least in the educational domain, and hence limit the generalizability of our results.

The British speakers all belong to the 16–25 year age group and were secondary school students from the Cambridge area (IViE; Grabe & Post, 2002) or were students at the University of Cambridge identified as speakers of Southern Standard British English (DyViS; Nolan et al., 2006). The Indian speakers were under- and postgraduate students at universities in Hyderabad and between 20 and 28 years of age. Five of them each spoke Hindi or Bengali (both Indo-European) as well as Telugu or Malayalam (both Dravidian), respectively, as first languages, which was defined as the principle language spoken at home during childhood and determined through a sociolinguistic interview. These two pairs of languages are the most widely spoken languages in India belonging to these two language families. All except one of the speakers went to English-medium schools from primary school up until college. With one exception, none of the speakers had spent an extended period of time abroad. They were all highly proficient in English, which was their dominant academic language and can be classified as speakers of Educated IndE (R. Fuchs, 2016: 105).

**Table 2.** Number of speakers across different age, gender, and ethnic groups in the Trinidadian dataset.

		Age Group^5^			Total Gender/Ethnicity
		16–25 Yr.	26–45 Yr.	46–65 Yr.	
Female	Afro-Trini	15	6	2	23
	Indo-Trini	2	3	0	5
	Mixed	7	4	1	12
	Other	2	2	1	5
Male	Afro-Trini	6	2	1	9
	Indo-Trini	3	1	0	4
	Mixed	6	0	1	7
	Other	4	0	0	4
Total Ethnicity	Afro-Trini	21	8	3	32
	Indo-Trini	5	4	0	9
	Mixed	13	4	2	19
	Other	6	2	1	9
Total Age		45	18	6	69

### 5.2 F0 extraction and parameters

Previous research has mostly relied on two broad approaches for the measurement of pitch level and range (see Patterson, 2000 for an overview): first, an analysis of pitch range as linked to specific phonological tones in the pitch contour (e.g., Mennen et al., 2012) and, second, an examination of overall distributional characteristics of F0 (e.g., Keating & Kuo, 2012; Leung & Deuber, 2014).

Our analysis focuses on the latter, namely the global distribution of F0, to allow for comparisons with the only previous acoustic study of pitch level and range in TrinE (Leung & Deuber, 2014). We follow recent studies on other New Englishes (R. Fuchs, 2018; Maxwell et al., 2018) in measuring three aspects of the global distribution of F0: (1) pitch level, (2) pitch range, and (3) pitch dynamism. A focus on these three global F0 parameters ensures comparability with previous findings for BrE and IndE.

Using pitch floors and ceilings appropriate for male (75 and 300 Hz) and female (100 and 500 Hz) speakers, we extracted F0 at 5 ms time steps over voiced stretches of speech with Praat,^6^ and computed all F0 parameters separately for both speaking styles for all speakers (the Praat script is available from the open science framework (OSF) website associated with this study: https://osf.io/94dbv/). Pitch level (or height) was quantified as the median of the F0 distribution in Hz (rather than the mean) to limit the confounding effects of potential outliers in the F0 distribution or measurement errors. For the same reason and, additionally, to account for the non-linear perception of F0 ranges in different frequency regions (Patterson, 2000: 42), pitch range (or span) was measured as the difference between the 90th and 10th percentile in semitones (80% range; see e.g., Busà & Urbani, 2011; Mennen et al., 2012 for other studies using these metrics). Pitch dynamism (or overall variability of pitch / coefficient of variation) was operationalized using the pitch dynamism quotient (pdq) (Hincks, 2004; see e.g, Cheng, 2020; Lee & Van Lancker Sidtis, 2017 for other studies using this metric), defined as the standard deviation of the F0 distribution of a given speaker divided by its mean in Hz.

### 5.3 Analysis

The statistical analysis of cross-varietal differences in pitch level, range, and dynamism between BrE, IndE, and TrinE is two-fold:

(1) Using linear regression models—one for each F0 parameter in each speaking style— that include gender as a predictor, we test for overall effects of variety on the investigated F0 parameters, that is, for differences between the varieties. Gender, as stated by the speakers, is included to ensure that overall differences are a result of global varietal and not possibly physiologically conditioned sex effects (which cannot in all cases be corrected for by normalization). The latter include previously observed differences in pitch level and range between female and male speakers (e.g., Pépiot, 2014). Such a control of confounding gender effects is also important because gender-based differences are not of a purely physiological, but also sociolinguistic nature. Additionally, given that (1) the degree of female-male differences in pitch range is not necessarily stable across different languages (Pépiot, 2014) or, by extension, different varieties, and (2) males and females are not equally distributed across our BrE, IndE, and TrinE datasets, we also investigate between-variety effects across both genders by including an interaction term for both in the modeling (variety × gender). It should be noted, however, that this modeling strategy does not allow teasing apart the extent of physiological (i.e., purely sex-based) and sociolinguistic gender effects. More specialized research designs, particularly designs combining acoustic and articulatory approaches that specifically measure and investigate sex-based anatomical differences and their effects on F0 variation (e.g., S. Fuchs & Toda 2010), may be required for this purpose. Pairwise comparisons of the estimated means in the models are based on Bonferroni-corrected *p*-levels. The regression analysis of cross-varietal differences is complemented by an inspection of intra-varietal variation and individual speaker differences (see below for further details).
(2) We investigate how consistently speakers who have a large/small pitch range also have a high/low degree of pitch dynamism across the different varieties. That is, we examine if and to what degree there are any between-variety differences in the co-variation of (large/small) pitch range and (high/low) pitch dynamism. To this end, Pearson cross-correlation coefficients are computed. Additionally, we compare the co-variation patterns across varieties visually in two-dimensional pitch range-pitch dynamism scatter plots, while taking into account gender-based (co-)variation and individual speaker differences.

The analysis of sociolinguistic variation in prosody in the TrinE dataset focuses on the sociolinguistic variables discussed above (Section 3) and laid out in our hypotheses (Section 4), namely gender, age, and ethnicity (and selected interactions). In view of Trinidad’s general sociolinguistic profile, the specific domain investigated here, and the amount of data collected (Section 5.1), additional sociolinguistic variables under scrutiny include the following: first, rurality, that is, whether a speaker is from an urban or rural area, as this variable has been shown to influence variation on the segmental phonological level, with rural speakers generally tending to produce more (stigmatized) Creole pronunciations than urban speakers (Leung, 2013; Winford, 1972, 1978). Although Gooden and Drayton (2017) report few urban–rural differences in intonational patterns in TEC, the variable rurality was included to investigate potential urban–rural differences —possibly in line with those at the segmental phonological level—in standard TrinE prosody, bearing in mind that patterns of variation in TEC and TrinE need not necessarily run in parallel (see Section 2). However, considering that the number of speakers from rural areas was limited (*n* = 5), urban–rural differences are only investigated in an exploratory manner, primarily intended to inform future research on sociolinguistic variation in TrinE prosody. Second, the variable prestige school was included since previous research on segmental phonological variation in the secondary education sector in Trinidad has shown that speakers affiliated with prestige schools—both students and teachers—show different realizations of some vowels compared to speakers associated with non-prestige schools (Meer, 2019; see also observations reported in Ferreira & Drayton, 2017). Third, it was investigated whether speakers that had lived abroad showed any differences in pitch level, range, or dynamism, considering that this group forms an essential part of users of standard English in the Trinidadian school domain and Trinidadian society more generally (see Section 5.1). Given that only two speakers were located in the south, north-south differences in the sample are not further explored in the present study. Table 3 summarizes all sociolinguistic variables, their levels, and the selected interactional effects under investigation.

**Table 3.** Sociolinguistic variables under investigation and their levels.

Sociolinguistic Effects	Levels
gender	Female, Male
age	Younger (16–25 Years), Middle (26–45 Years), Older (46–65 Years)^7^
ethnicity	Afro-, Indo-, Mixed-identifying Trinidadian, Other
gender × ethnicity	
age×ethnicity	
rurality	Urban, Rural
prestige school	Prestige School, Non-prestige School
lived abroad	Yes, No

Sociolinguistic effects are tested for significance via mixed-effects linear regression models – one for each F0 parameter in each speaking style—that include school as a random intercept to account for random variation due to the fact that speakers were unequally and randomly clustered in and sampled from different schools. By-school random slopes are not included because the model building process showed that the data did not support a more complex random effects structure: models with by-school random slopes for any of the fixed effects failed to converge, even when the number of maximum iterations was increased or random (intercept-slope) correlation parameters were set to zero to simplify the random effects structure (following recommendations in Barr et al., 2013: 276; Bates et al., 2015: 4; Matuschek et al., 2017).^8^ All sociolinguistic predictors, including gender, are modeled as fixed effects.^9^ The models are followed by Bonferroni-adjusted pairwise comparisons of the estimated means. The mixed-effects structure, combined with the adjusted pairwise comparisons, allows for conservative control of possible Type I errors. Given that conservative models are run, effects close to statistical significance are also reported in an attempt to minimize potential Type II errors.

Separate models are fitted for each F0 parameter in each speaking style—rather than for pooled data including both styles—to optimize the modeling of the specific data at hand. Firstly, previous analyses of pitch level, range, and dynamism in the IndE and parts of the BrE datasets (R. Fuchs, 2018) showed considerable between-style differences not only in terms of measures of central tendency but also the degree of variation in the F0 parameters (heterogeneity of variance), which can pose problems for linear modeling (e.g., Field, 2018: 237-239). Secondly, fitting models based on pooled data would result in a considerably more complex model structure with a large number of two-way and three-way interaction terms including style (one additional interaction term for each of the predictors and one additional main effect for style). Unlike for the predictors per se (such as those in Table 3), however, due to the scarcity of previous research, we were unable to specify individual hypotheses for each of the various interactions with style that would have lent themselves to focused null hypothesis testing. Additionally, these models would include a high number of pairwise comparisons for the interaction effects. Given that the alpha level is (Bonferroni-)adjusted for the number of these comparisons, such a procedure would likely result in too conservative control of Type I error and thus potential loss of statistical power. We acknowledge that the chosen modeling strategy means that we cannot straightforwardly compare between-style differences across varieties and social groups. However, given that the investigation of between-style differences in F0 parameters is not one of our primary aims, the analyses in the current study focus on *within*-style differences between varieties and social groups, which is not an uncommon modeling strategy in studies comparing variation in fundamental frequency across speaking styles (e.g., Keating & Kuo, 2012).

All best-fit linear models in the current study were built in SPSS 26 in a manual backward elimination process (Twisk 2006: 82; Zuur et al., 2009: 121–122), starting with a full model including the above specified main effects and interactions. Model selection was informed by the two χ^2^ likelihood parameters Akaike information criterion (AIC) and Bayesian information criterion (BIC; Kuha, 2004; Vandekerckhove et al., 2015: 306)^10^ and our hypotheses derived from previous research (Gelman & Hill, 2007: 69). All models rely on the maximum likelihood estimation method (Twisk, 2006: 29), with degrees of freedom generated using Satterthwaite approximation. The factor speaker is not included as a random effect, given that each regression model only contains a single datapoint for each speaker (see Barr et al., 2013: 275). Levels of significance of the Bonferroni-adjusted pairwise comparisons of the estimated means are displayed as follows: *p* < .05 (*), *p* < .01 (**), *p* < .001 (***).

In the following sections, we report the means (and confidence intervals) estimated by the linear models for each fixed effect rather than observed means in an attempt to control for gender and other confounding effects. That is, each fixed effect is considered in *ceteris paribus* conditions while holding other (confounding) fixed and random effects statistically constant. Additionally, we show and analyze the observed distributions of the data and individual speaker differences across the different F0 parameters as a way to scrutinize variation within TrinE, BrE, and IndE as well as different social groups in TrinE beyond measures of central tendency. Visualizations of the data take these different aspects into account. This combined approach allows us to inspect more fine-grained patterns of variation and change in TrinE prosody.

## 6 Results

### 6.1 Cross-varietal comparison of pitch variation

**Figure 1.**
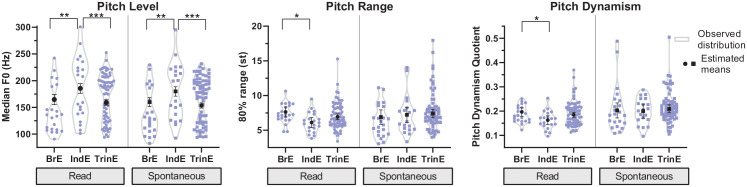
Estimated means and confidence intervals superimposed on observed distributions (smoothed violin plots with individual values) for pitch level (median F0 in Hz), range (80% range in semitones), and dynamism (pitch dynamism quotient) across British, Indian, and Trinidadian English in read and spontaneous speech (effect: variety). Levels of significance: *p* < .05 (*), *p* < .01 (**), *p* < .001 (***).

A series of linear models revealed that the varieties, while controlling for gender, differ significantly from each other in the global distribution of F0, but to different degrees depending on the F0 parameter in question and the speaking style (see Figure 1). For pitch level, we find significant overall effects of variety in both read, *F*(2, 111) = 12.94, *p* < .001, and spontaneous speech, *F*(2, 111) = 13.33, *p* < .001, with similar inter-varietal differences for both speaking styles. Bonferroni-corrected pairwise comparisons of the estimated means show that TrinE has a significantly lower average pitch level than IndE in both read (159 Hz vs. 186 Hz, *p* < .001) and spontaneous speech (154 Hz vs. 180 Hz, *p* < .001). The average pitch level in TrinE is also slightly lower than in BrE (read: 165 Hz, spont.: 160 Hz), but these differences did not reach significance. The observed distribution of median F0 across speakers of the three varieties in both speaking styles is generally similar and demonstrates expected gender-based differences in pitch level, as shown by the bimodal shape of the violin plots for pitch level in Figure 1.

Significant differences between varieties in pitch range were only observed in read speech, *F*(2, 111) = 4.44, *p* < .05. BrE has the widest mean pitch range in read speech (7.6 st), followed by TrinE (6.9 st) and IndE with the smallest mean range (6.1 st). However, while BrE has a significantly higher 80% range than IndE (*p* < .05), differences between TrinE and both BrE and IndE are not significant. In spontaneous speech, a different pattern can be observed: TrinE (7.4 st) has a marginally wider mean pitch range than both IndE (7.2 st) and BrE (6.9 st), but these differences are not significant. The distributions of the data parallel these central tendencies but show an additional trend: several Trinidadian speakers have very wide pitch ranges (> 9 st) in both speaking styles, particularly in spontaneous speech (read: *n* = 9; spont.: *n* = 14), while this number is substantially lower for speakers of IndE and especially BrE.

The findings for pitch dynamism are generally in line with those for pitch range. A significant overall effect of variety on pitch dynamism was found in read, *F*(2, 111) = 3.69, *p* < .05, but not in spontaneous speech. In read speech, on average, pitch in BrE is most dynamic (pdq = 0.197), followed by TrinE (pdq = 0.185) and IndE (pdq = 0.163), with only the BrE-IndE difference reaching significance (*p* < .05). In spontaneous speech, TrinE (pdq = 0.209) has a slightly higher mean pitch dynamism quotient than both BrE (pdq = 0.203) and IndE (pdq = 0.200), but none of these differences reached statistical significance. The distribution of pdq scores across speakers shows a tendency similar to that observed for pitch range: compared to BrE and especially IndE, there is a considerably higher number of Trinidadian speakers with very dynamic pitch (pdq > 0.25) in read and, in particular, spontaneous speech (read: *n* = 7; spont.: *n* = 14).

**Figure 2.**
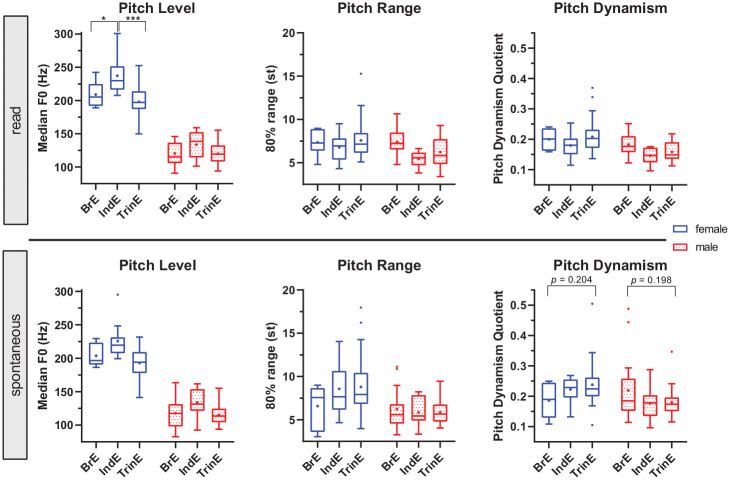
Observed distribution (Tukey boxplots with means as dots and medians as lines) for pitch level, range, and dynamism across female and male speakers of British, Indian, and Trinidadian English in read and spontaneous speech (effect: variety × gender). Levels of significance: *p* < .05 (*), *p* < .01 (**), *p <* .001 (***).

Overall gender effects are controlled for in the above regressions, but inter-varietal differences also exist within-gender groups. We therefore additionally analyze pitch level, range, and dynamism as a function of the interaction of variety and gender. We present the F0 data in the form of boxplots (see Figure 2) because these allow us to zero in on the variety-gender-specific percentile distributions in addition to central tendencies. We here focus and report on larger within-gender differences that provide insights for the inter-varietal comparison.

In read speech, an interaction effect of variety and gender was found on pitch level that was close to statistical significance, *F*(2, 111) = 3.06, *p* = .051. Among female speakers, TrinE has a significantly lower mean pitch level than IndE (198 Hz vs. 237 Hz, *p <* .001), and there is relatively little overlap across the distributions. Although the difference in mean pitch level to BrE (209 Hz) is not significant, approximately 25 percent of the female TrinE speakers (the lowest quartile in the TrinE boxplot) have a median F0 below the minimum pitch level of the BrE data. Inter-varietal differences in pitch range and pitch dynamism within-gender groups are not significant and rather small. There are, however, a few female Trinidadian speakers with higher pitch range and dynamism scores compared to BrE and particularly IndE, as indicated by the differences in length of the upper whiskers of the boxplots. The male TrinE speakers tend to have somewhat lower pitch range and dynamism values than the male BrE and slightly higher scores than the male IndE speakers.

There are more notable inter-varietal differences within-gender groups in spontaneous speech. These differences, however, do not necessarily concern measures of central tendency (mean and median) by themselves. These were mostly found to be similar and not to differ significantly from each other, although a significant interaction of variety and gender was observed for pitch dynamism, *F*(2, 111) = 3.37, *p* < .05.^11^ Instead, most inter-varietal differences in spontaneous speech regard the total distribution of the data:

(1) In contrast to female BrE and IndE speakers, approximately half of all female TrinE speakers have F0 levels below 200 Hz; unlike in female British and Indian speakers, pitch level in these female speakers approximates the pitch level distribution of male speakers.
(2) While male speakers across the three varieties generally show few differences in pitch range and dynamism, especially in terms of measures of central tendency, a larger proportion of male BrE speakers has very dynamic pitch compared to male TrinE speakers, that is, pdq scores > 0.25.
(3) Substantial inter-varietal differences exist among female speakers. More than 25 percent of all female TrinE (and IndE) speakers have a very wide pitch range > 10 st, whereas none of the female BrE speakers has such a wide range. For pitch dynamism, a similar tendency can be observed. More than half of the female BrE speakers have pdq scores < 0.2; in TrinE, in contrast, around 75 percent of all females have pdq scores > 0.2. The difference in pitch dynamism compared to IndE is not as large. However, while in IndE there are almost no speakers with very high pdq scores > 0.25, approximately 25 percent of all TrinE female speakers have pdq scores higher than this threshold.

**Figure 3.**
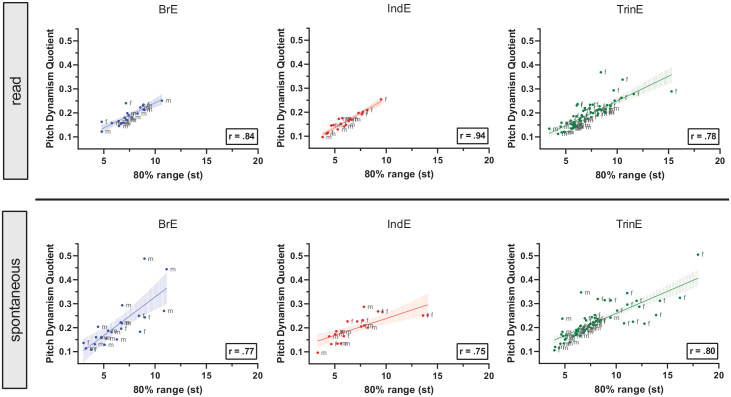
Co-variation of pitch range and pitch dynamism in female and male speakers across British, Indian, and Trinidadian English in read and spontaneous speech: points for individual speakers, point labels providing gender information, linear regression lines with shaded 95% confidence intervals, and Pearson correlation coefficients.

The simultaneous analysis of pitch range and pitch dynamism in the sample shows that there are similarities in the overall co-variation pattern of these F0 parameters across BrE, IndE, and TrinE, but also some TrinE-specific tendencies (see Figure 3). Across varieties, both in read and spontaneous speech, significant positive correlations, all with Pearson correlation coefficients above 0.75 (each at *p* < .001), indicate similarly overall strong relationships between pitch range and dynamism. That is, speakers who have a wide/narrow pitch range also tend to have a high/low degree of pitch dynamism. This finding is not unexpected, considering that pitch range and dynamism measure related aspects of the overall variability of F0. Small differences exist in the regression slopes across varieties. While these are relatively similar in read speech (BrE: 0.021, IndE: 0.024, TrinE: 0.021), slightly larger differences can be observed in spontaneous speech (BrE: 0.032, IndE: 0.014, TrinE: 0.018). The latter indicate that, in spontaneous speech, Trinidadian speakers with a wide pitch range tend to have slightly higher pdq scores relative to Indian and slightly lower pdq scores than British speakers with the same pitch range. Visual inspection of the pitch range-pitch dynamism scatterplots further shows distributional trends specific to TrinE:

(1) There are substantially more speakers with both a wide pitch range (> 9 st) and very dynamic pitch (pdq > 0.25) in TrinE than in BrE and IndE, especially in spontaneous speech.
(2) These speakers are almost exclusively female in TrinE, but do not show a large degree of gender-stratified co-variation in BrE and IndE.
(3) Within TrinE, this gender-stratified pattern is more pronounced in spontaneous than read speech. In spontaneous speech, a gender split exists in the data, with female Trinidadian speakers having consistently higher pitch range and dynamism scores than Trinidadian males.
(4) Among TrinE speakers that either have a large pitch range (> 9 st) or very dynamic pitch (pdq > 0.25), relatively variable co-variation can be observed.

### 6.2 Sociolinguistic variation in TrinE prosody

Having explored differences in pitch level, range and dynamism between TrinE, IndE and BrE, we now focus on sociolinguistically conditioned variation in these variables within TrinE.

#### 6.2.1 Statistical modeling

Linear mixed-effects models revealed that variation in TrinE prosody is sociolinguistically conditioned by a variety of different factors (see Table 4 for an overview of the best-fit models). Significant main effects were found for gender, age, rurality, and ethnicity with regard to all F0 parameters, respectively, but not necessarily in both speaking styles. Additional significant effects include the two-way interactions of gender × ethnicity (only in the case of pitch level) and age × ethnicity (only in the case of pitch dynamism). That is, there are significant ethnic differences in pitch level within male and female speaker groups, and ethnic differences in pitch dynamism vary significantly across age groups. No significant main effects were found for lived abroad and prestige school, and descriptive data analysis further showed that almost no differences exist between speakers that have (not) lived abroad or are (not) affiliated with a prestige school across all F0 parameters in both speaking styles. A distributional exception to this general tendency is that speakers associated with prestige schools do not have very high pitch range (> 10 st) and dynamism scores (pdq > 0.3) in spontaneous speech, while many speakers from non-prestige schools have pitch range and dynamism scores above these thresholds (range: 40%, dynamism: 25% of speakers).

**Table 4.** Summary of best-fit mixed-effects linear regression models for pitch level, range, and dynamism in read and spontaneous Trinidadian English. Levels of significance: *p* < .05 (*), *p* < .01 (**), *p* < .001 (***).

	Level (read)				Range (read)				Dynamism (read)			
	*dfn*	*dfd*	*F*	*p*	*dfn*	*dfd*	*F*	*p*	*dfn*	*dfd*	*F*	*p*
Intercept	1	69	1151.9	***	1	69	603.1	***	1	69	706.4	***
gender	1	69	188.1	***	1	69	9.4	**	1	69	20.5	***
age	2	69	4.7	*	2	69	3.2	*	2	69	6.9	**
rurality	1	69	8.8	**								
ethnicity	3	69	4	*					3	69	1.8	n.s.
gender×ethnicity	3	69	3.3	*								
age×ethnicity									4	69	2.1	.089
	Level (spont.)				Range (spont.)				Dynamism (spont.)			
	*dfn*	*dfd*	*F*	*p*	*dfn*	*dfd*	*F*	*p*	*dfn*	*dfd*	*F*	*p*
Intercept	1	69	1460	***	1	69	206.3	***	1	69	301.7	***
gender	1	69	180.6	***	1	69	23.1	*	1	69	16.1	***
age					2	69	5.3	**	2	69	5.9	**
rurality	1	69	5.7	*	1	69	8.5	**	1	69	6.3	*
ethnicity	3	69	3.7	*	3	69	2.2	0.099	3	69	3.8	*
gender×ethnicity	3	69	4.9	**								
age×ethnicity									4	69	2.3	.068

Detailed descriptions of sociolinguistic variation in the F0 parameters in both speaking styles are presented in Sections 6.2.2–6.2.7. The analysis is structured according to the significant sociolinguistic effects identified in Table 4.

#### 6.2.2 Gender

In line with the descriptive account of the gender-stratified distribution of the TrinE data above (see Section 6.1 for details), significant effects of gender were found for all F0 parameters and across both speaking styles: gender explains a substantial proportion of the sociolinguistic variation found in the Trinidadian data (see Table 5 for an overview). On average, females have significantly higher pitch levels (read: 55.7%, spont.: 52.5% higher), significantly wider pitch ranges (read: 20.6%, spont.: 34.4% wider), and significantly more dynamic pitch (read: 26.3%, spont.: 23.3% more dynamic).

**Table 5.** Estimated means for female and male Trinidadian speakers for pitch level, range, and dynamism in read and spontaneous speech (effect: gender). Levels of significance: *p* < .05 (*), *p* < .01 (**), *p* < .001 (***).

		female (*n* = 45)	male (*n* = 24)	*P*
Level	Read	200 Hz	129 Hz	***
	Spontaneous	196 Hz	128 Hz	***
Range	Read	8 st	6.7 st	**
	Spontaneous	10.5 st	7.8 st	***
Dynamism	Read	.221	.175	***
	Spontaneous	.275	.223	***

#### 6.2.3 Age

The analysis showed that significant differences exist between age groups across all F0 parameters and both speaking styles, except for pitch level in spontaneous speech. As regards pitch level, a significant overall effect was only found in read speech: speakers in the youngest age group (< 26 years) have the highest median F0 (174 Hz), followed by the middle-aged group (< 46 years; 162 Hz) and the oldest group with the lowest level (158 Hz). It is, however, noteworthy that the Bonferroni-adjusted pairwise comparisons between younger and middle-aged (*p* = .051), younger and older (*p* = .093), and middle-aged and older (*p* = 1) are only close to significance, if at all.

**Figure 4.**
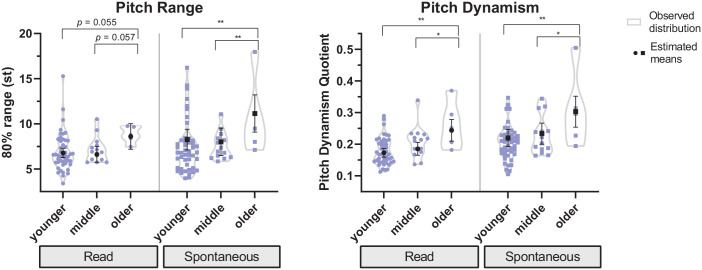
Estimated means and confidence intervals superimposed on observed distributions (smoothed violin plots with individual values) across age groups (< 26 years/younger, < 46 years/middle, < 66 years/older), in Trinidadian English: pitch range and dynamism in read and spontaneous speech (effect: age). Levels of significance: *p* < .05 (*), *p* < .01 (**), *p* < .001 (***).

Differences between age groups with regard to pitch range and pitch dynamism show a more pronounced and relatively straightforward trend in both speaking styles (see Figure 4). That is, globally speaking, pitch range tends to be significantly wider in the oldest than in the two youngest age groups, while differences between younger and middle-aged speakers are small on average and their estimated means not significantly different from each other. The distribution of the entire data across age groups (see the shape of the violin plots in Figure 4) indicates further that, among the youngest age group, there is a considerable number of speakers who have narrower pitch ranges and less dynamic pitch than speakers in the intermediate age group. For pitch dynamism, a similar distributional tendency can be observed in both speaking styles. At the same time, there are a few individual speakers in the younger (and middle-aged) group with wide pitch ranges and relatively dynamic pitch (in spontaneous speech).

Specifically, the following differences between the estimated means can be identified: with regard to pitch range in read speech, older speakers have a wider mean range (8.6 st) than younger (*p* = .055) and middle-aged speakers (6.8 st vs. 6.6 st, *p* = .057), but these differences are only close to significance. Larger and significant differences in pitch range between age groups were found in spontaneous speech, with older speakers (11.2 st) having a wider mean pitch range than the intermediate (8.3 st, *p* < .01) and the youngest age group (8 st, *p* < .01). Regarding pitch dynamism, estimated mean pdq scores decrease with age group both in read (pdq = 0.244 vs. 0.185 vs. 0.173) and in spontaneous speech (pdq = 0.303 vs. 0.234 vs. 0.220; see Figure 4 for the individual *p*-levels of the pairwise comparisons).

#### 6.2.4 Rurality

**Figure 5.**
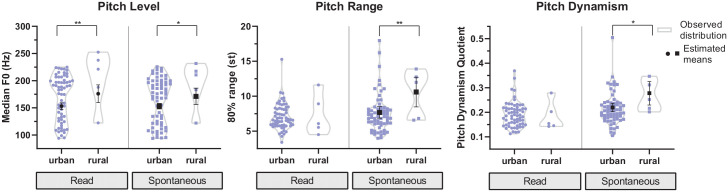
Estimated means and confidence intervals superimposed on observed distributions (smoothed violin plots with individual values) for Trinidadian speakers located in urban and rural areas: pitch level, range, and dynamism in read and spontaneous speech. Non-significant overall effects not displayed (effect: rurality). Levels of significance: *p* < .05 (*), *p* < .01 (**), *p <* .001 (***).

Differences between urban and rural speakers exist across all three F0 parameters (see Figure 5). Rural speakers have a significantly higher mean pitch level than urban speakers, both in read (176 Hz vs. 153 Hz, *p* < .01) and in spontaneous speech (171 Hz vs. 153 Hz, *p* < .05). rurality effects on pitch range and dynamism were only observed in spontaneous speech, where rural speakers were shown to have significantly wider mean pitch ranges (10.6 st vs. 7.7 st, *p* < .01) and use, on average, more dynamic pitch (pdq = 0.278 vs. 0.220, *p* < .05) than urban speakers. Similar differences in pitch range or dynamism were not found in read speech. Despite these overall differences between urban and rural speakers in pitch range and dynamism in spontaneous speech, there are also a few urban speakers that have similar pitch range and dynamism values compared to most rural speakers.

#### 6.2.5 Ethnicity

**Figure 6.**
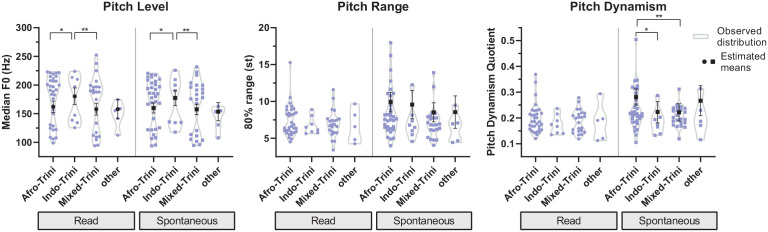
Estimated means and confidence intervals superimposed on observed distributions (smoothed violin plots with individual values) across different Trinidadian ethnic groups: pitch level, range, and dynamism in read and spontaneous speech. non-significant overall effects not displayed (effect: ethnicity). Levels of significance: *p* < .05 (*), *p* < .01 (**), *p* < .001 (***).

Mixed-effects regression analysis revealed differences in the F0 parameters across ethnic groups (see Figure 6). While these are overall significant for pitch level in read and spontaneous speech and pitch dynamism in the latter speaking style, the effect of ethnicity on pitch range in spontaneous speech is only close to significance (*p* = .099). With regard to pitch level, it was found that Indo-Trinidadians, on average, have the highest median F0 (read: 180 Hz, spont.: 177 Hz), with both Afro- (read: 162 Hz, spont.: 160 Hz) and mixed-identifying Trinidadians (read: 158 Hz, spont.: 158 Hz) having significantly lower levels (see Figure 6 for the individual *p*-levels of the pairwise comparisons). A different pattern was observed for pitch dynamism and range in spontaneous speech: Afro-Trinidadians have significantly more dynamic pitch (pdq = 0.281) than Indo- (pdq = 0.223, *p* < .05) and mixed-identifying Trinidadians (pdq = 0.222, *p* < .01). A similar ranking can be found for pitch range, with Afro-Trinidadians (9.9 st) having (non-significantly) larger average ranges than Indo- (9.6 st) and particularly mixed-identifying speakers (8.5 st). The distribution of the data across ethnic groups and F0 parameters is relatively similar and reflects these central tendencies for pitch level, range, and dynamism (notwithstanding the fact that a few individual Afro-Trinidadian speakers have relatively high range and dynamism scores in spontaneous speech). Differences for read speech are not significant and marginal.

#### 6.2.6 Gender and Ethnicity

A significant interaction effect of gender and ethnicity was found for pitch level in read and spontaneous speech. Similar to overall ethnic differences independent of gender, female and male Indo-Trinidadian speakers have consistently higher estimated mean pitch levels than female and male speakers from other ethnic groups, respectively (see Table 6). A comparison across gender groups shows that average differences across Afro-, Indo-, and mixed-identifying Trinidadians for both speaking styles are slightly bigger for male (read: Δ_Level_ = 19 Hz, spont.: Δ_Level_ = 17 Hz) than female speakers (read: Δ_Level_ = 11 Hz, spont.: Δ_Level_ = 10 Hz ). At the same time, there is more variation in pitch level in female than male Afro-Trinidadian speakers. A considerable number of female Afro-Trinidadians have substantially lower pitch levels compared to female Indo-Trinidadians, while this difference is smaller in scope in the male group: the difference in minimum pitch level found between the Afro- and Indo-Trinidadian group is considerably larger among female (read: Δ_Level_ = 47 Hz, spont: Δ_Level_ = 49 Hz) than male speakers (read: Δ_Level_ = 27 Hz, spont: Δ_Level_ = 24 Hz).

**Table 6.** Estimated mean pitch levels for female and male Trinidadian speakers across different ethnic groups in read and spontaneous speech (effect: gender×ethnicity). Levels of significance: *p* < .05 (*), *p* < .01 (**), *p* < .001 (***).

		Female				Male				*P*
		Afro (*n* = 23)	Indo (*n* = 5)	Mixed (*n* = 12)	Other (*n* = 5)	Afro (*n* = 9)	Indo (*n* = 4)	Mixed (*n* = 7)	Other (*n* = 4)	
Level (Median F0 in Hz)	Read	204	219	202	177	121	142	114	139	*
	Spont.	201	215	200	167	119	140	115	140	**

Ethnic differences for pitch range and dynamism within male and female speaker groups are not significant and are overall small—both with regard to measures of central tendency and the distribution of the data. A minor difference between ethnic groups is that around 25 percent of the female Afro-Trinidadian speakers have rather low pitch range (< 7.5 st) and dynamism (pdq < 0.2) scores in spontaneous speech compared to female speakers in other ethnic groups.

#### 6.2.7 Age and ethnicity

In addition to the main effects of age and ethnicity, variation in pitch dynamism is explained by a significant interaction of both factors, both in read and in spontaneous speech. That is, ethnic groups differ from each other to different degrees depending on their age. In order to quantify inter-ethnic differences depending on their age, we compare the mean accumulated difference in pitch dynamism between Afro-, Indo-, and mixed-identifying Trinidadians across the three age groups (see Table 7). While older speakers, both in read and in spontaneous speech, show the largest inter-ethnic differences in pitch dynamism, differences between the three ethnic groups are smaller in the intermediate age group, and smallest in the youngest group of speakers. The distribution of the data across different age and ethnic groups reflects these accumulated differences; no diverging tendencies were observed. No significant interaction effects were found concerning pitch level and pitch range, with differences across ethnic and age groups being small.

**Table 7.** Estimated mean accumulated difference in pitch dynamism (Δ_pdq_) between Afro-, Indo-, and Mixed-identifying Trinidadians across different age groups (effect: age × ethnicity).^12^

	Pitch Dynamism	
	Read	Spontaneous
older (< 66 years; *n* = 6)	0.072	0.113
middle (< 46 years; *n* = 18)	0.022	0.044
younger (< 26 years; *n* = 45)	0.007	0.013

## 7 Discussion

Based on read and spontaneous data from 111 speakers (69 from Trinidad, 22 from England, and 20 from India), this study has analyzed pitch level, range, and dynamism in TrinE in comparison to BrE and IndE, and investigated sociolinguistic variation in TrinE prosody with a view to these global F0 parameters.

With regard to the cross-varietal comparison of pitch variation in terms of level, range, and dynamism (RQ1), our hypotheses (H1–H3; see Section 4) can only partially be confirmed. As regards similarities in pitch level (H1), our hypothesis can be confirmed to the extent that pitch level in TrinE was not found to be higher than in BrE and IndE; compared to both varieties, TrinE generally has the lowest level. While differences to IndE are larger, differences to BrE were observed to be small both in read and spontaneous speech. This finding is in contrast to anecdotal evidence claiming that Trinidadian speech is characterized by a particularly high pitch level (Winer, 1993: 20). The assumption that TrinE has a wider pitch range than other Englishes (H2) can also only partially be confirmed: TrinE has a (non-significantly) wider pitch range than IndE but a somewhat smaller range than BrE in read speech, while in spontaneous speech it has a (non-significantly) wider range than both BrE and IndE. Our assumption of TrinE having exceptionally dynamic pitch (H3) can also only partially be confirmed from an overall perspective. TrinE has (non-significantly) more dynamic pitch than IndE in read speech, but not BrE, while TrinE only has marginally (and non-significantly) more dynamic pitch than IndE and BrE in spontaneous speech. In sum therefore, TrinE does not have a wider pitch range (with the exception of pitch range in spontaneous speech) or more dynamic pitch than the other two varieties on average.

However, an analysis of the influence of speaker gender complicates the picture. Male speakers across the three varieties generally show few differences in pitch range and dynamism, and these differences are below the level of significance. In contrast, considering the entire distributions of the data (in addition to measures of central tendency, which also show few significant overall differences), considerably more female TrinE speakers were found to have a wider pitch range than female BrE speakers in spontaneous speech and more pitch dynamism than both female BrE and IndE speakers in this speaking style. The co-variation analysis also revealed that more female TrinE speakers have both a wide pitch range and very dynamic pitch compared to their counterparts in BrE and IndE. To some extent, these differences in the distributions of the data may also be the result of a particular group of female speakers with very high pitch range and/or dynamism. The analysis of co-variation of pitch range and dynamism indeed suggests that, in contrast to BrE and IndE, there are some female speakers that have very dynamic pitch, but within a generally smaller pitch range, while others produce a wide range but considerably less pitch variability within this range. Consequently, despite the hypotheses not being valid for TrinE overall, H2 and H3 can be confirmed for most female TrinE speakers: it is female Trinidadian speakers, in spontaneous speech, who have particularly wide pitch ranges and dynamic pitch. Our findings on TrinE prosody are thus partially in line with anecdotal evidence claiming that TrinE (as well as other Caribbean varieties of English more generally) have a wider pitch range than other Englishes (Winer, 1993: 20; see also Wells, 1982: 573–575). The results also confirm Wilson’s (2007) first observations of TrinE having a wider pitch range than BrE but show that this is not necessarily the case for Trinidadian speakers across the board.

The findings thus emphasize the influence of sociolinguistic variation in TrinE prosody and how it may account for TrinE “sing-song” (RQ2). As hypothesized (H4), we find that pitch variation in TrinE is not homogenous, but rather systematically conditioned by different sociolinguistic variables. In line with previous research on TrinE (Leung & Deuber, 2014), the analysis revealed that variation in pitch in TrinE can in part be explained by the sociolinguistic variables gender and ethnicity and their interaction. Moreover, similar to findings observed for Trinidadian Creole (Gooden & Drayton, 2017), variation in standard TrinE is also conditioned by a speaker’s age and the interaction of the factors age and ethnicity.

As regards the kinds and degrees of influence these variables have on pitch variation in TrinE (H5a-d), the results reveal a complex and multifaceted picture of current variation and change in TrinE prosody. In line with the popular stereotype and previous research on ethnic differences in the global distribution of F0 (Leung & Deuber, 2014; H5a), Indo-Trinidadians were shown to have higher pitch levels than Afro- and mixed-identifying speakers. However, similar overall ethnic differences for pitch range and dynamism were not observed. Additionally, it was hypothesized that ethnic differences depend on speaker gender, with only female Indo-Trinidadians having higher levels (and wider ranges) (H5b; see Leung & Deuber, 2014). The analysis showed, however, that overall ethnic differences in pitch level (and range) are largely stable across both genders (contra findings in Leung & Deuber, 2014). A minor exception to this overall trend is the large degree of variation observed among female Afro-Trinidadians, with several speakers having comparatively low pitch levels, narrow ranges, and less dynamic pitch (partially in line with findings in Leung & Deuber, 2014). In sum, ethnic differences are largely restricted to pitch level, smaller for pitch range and dynamism, and mostly independent of gender. Potential explanations for these differences in findings between both studies could be related to a possible confounding influence from outliers given that Leung and Deuber (2014) used maximal range or simply the comparably smaller number of speakers analyzed there.

We also hypothesized that ethnic differences may be less important among younger speakers than older speakers, which may be due to convergence over time, similarly to what has been shown for TEC (Gooden & Drayton, 2017; H5c). In parallel to Gooden and Drayton’s (2017) observations for TEC, the observed age differences suggest an apparent-time change in pitch range and dynamism in TrinE, as indicated by measures of central tendency and distributional properties of the different age groups (although a certain degree of variability also exists in each of the age groups, especially the youngest one). However, while a possible change in progress can be inferred for both varieties, the results at hand point to an inverse trend in standard TrinE compared to TEC; there seems to be an apparent-time change toward less variability of pitch in younger speakers than older ones, that is, these speakers tend to have narrower pitch ranges and lower pdq scores (contra H5c). These findings may in fact complement each other and reveal a multifaceted change in progress in Trinidad’s current Creole continuum: as stated above (see Section 2), previous research has shown that standard English in Trinidad is not only subject of direct influence from TEC but may, in particular contexts, be defined negatively by its distance from Creole pronunciations (Deuber & Leung, 2013: 309). Features traditionally associated with TEC therefore often tend to be avoided in standard speech, particularly in formal contexts (e.g., Leung, 2013: 129; Meer, 2019; Wilson, 2014; see Irvine-Sobers, 2018 on standard Jamaican English). This underlying mental representation of standard TrinE (see Milroy, 2001: 543 on mental representations of standards) may lead speakers not only to avoid particular segmental phonological features of TEC when speaking standard TrinE (e.g., th-stopping or monophthongized and velarized realizations of down [dɔŋ]; see also Irvine-Sobers, 2018) but also prosodic aspects they associate with Creole, such as a high variability in pitch. As a result, when speaking standard TrinE, speakers may use a smaller pitch range and less dynamic pitch than is generally common in TEC. The fact then that TEC prosody has been undergoing a change in progress toward more consistent accentual phrasing and thus more frequent variation of high and low tonal targets and pitch variability, might thus explain why the opposite trend can be observed in TrinE.

Finally, we also find evidence of convergence between different ethnic groups in apparent time (H5d), similar to previous observations on a phonological level in TEC (Gooden & Drayton, 2017). Inter-ethnic differences in pitch dynamism in spontaneous speech were found to decrease in apparent time, resulting in only minor ethnic differences in pitch dynamism among younger speakers. Given the already very limited ethnic differences in pitch range and dynamism (but not level), our results suggest overall that a high degree of inter-ethnic convergence with a view to pitch variability has been reached in TrinE.

A further factor found to condition variation in standard TrinE prosody is the urban–rural divide, with rural speakers having higher pitch levels, wider ranges, and more dynamic pitch, mostly in spontaneous speech. The other sociolinguistic factors investigated here, time spent abroad and prestige school, were not significant. However, descriptive data analysis revealed that there is a tendency among prestige school speakers to avoid the use of a very wide pitch range and exceedingly dynamic pitch. In parallel to the apparent-time change observed in the data, the urban–rural divide could possibly be due to speakers attempting to distance themselves from what they may judge to be a characteristic prosodic aspect of Creole. While there are few differences in the intonational phonologies of urban and rural speakers in the production of TEC (Gooden & Drayton, 2017), urban speakers may avoid more variable pitch in the production of standard TrinE compared to rural speakers, who have generally been shown to retain more Creole-associated segmental features in their speech, including in more formal speaking styles (Leung, 2013; Winford, 1972, 1978). A similar explanation might hold for prestige school speakers avoiding very dynamic pitch patterns compared to speakers affiliated with other schools.

Although it is based on a large number of speakers overall, a limitation of the current study is the size of some of the sub-groups in the analysis of sociolinguistic variation in TrinE prosody. Specifically, few speakers were sampled from rural areas (*n* = 5) and the oldest age group (*n* = 6). The oldest age group, moreover, did not include any Indo-Trinidadian speakers. Our finding that TrinE prosody varies along age, urban–rural, and both age and ethnic lines should therefore be tested more explicitly for these specific sub-groups. Given that the data at hand show relatively clear overall distributional tendencies across the factors age, rurality, and ethnicity and differences between the sub-groups in question were both statistically significant and of a relatively high magnitude, the observed apparent-time changes and the urban–rural divide may be robust. Ultimately, however, further research with larger sample sizes for these individual groups is needed to confirm the effects observed in the analysis.

Another limitation of our analysis is the use of non-identical stimuli, which might have introduced task-dependent variability. Specifically, one protocol was followed to elicit the TrinE data, another to elicit part of the BrE data (drawn from the IViE corpus) and another to elicit the remainder of the BrE as well as the IndE data (DyViS stimuli). While the use of a single protocol for data elicitation would have avoided this potential variability, we submit that such resources for cross-varietal prosodic research, involving both read and spontaneous speech, are at present exceedingly rare. Moreover, the impact of this potential source of variability in the present study is limited since (1) the bulk of our results relate to internal variation in TrinE, for which data were elicited with a single protocol and (2) we can gage the extent of task-dependent variability in our data by examining the BrE data more closely, for which we have data from male speakers in the DyViS and IViE corpora. While none of the sentences in the DyViS passage were questions, 12% of the sentences in the IViE reading passage were. We observed no significant differences in pitch level, range, and dynamism between the male speakers in both corpora in read speech. However, we observed significantly higher pitch level, range and dynamism in the spontaneous male BrE IViE data compared to the DyViS speakers. This effect might be due to variations in the speaking task, as the dyadic peer conversations in IViE would have involved a small number of questions, whereas the DyViS police interview task required speakers mainly to respond to the interviewer’s questions. Still, even in the DyViS interview task speakers did occasionally ask questions. Future research on cross-varietal prosody should attempt to use identical tasks wherever possible. However, we submit that the use of not quite identical existing data holds great potential for cross-varietal prosodic research that would otherwise be left unexplored, given the enormous effort involved in collecting data from scratch for multiple populations.

## 8 Conclusion

Based on a cross-varietal comparison of pitch variation in TrinE and two other varieties of English (BrE and IndE), as well as an investigation into sociolinguistic variation in TrinE prosody, the current study provides a phonetic perspective on the extent to which a high degree of pitch variability may be considered an endonormative feature of TrinE at the level of speech production. Thus, the study also sheds light on the ways in which TrinE-specific pitch variation patterns may contribute to its distinct “sing-song” prosody.

Our findings suggest that a wide pitch range (but not a high degree of pitch dynamism) could potentially be considered an endonormative feature of TrinE that distinguishes it from other varieties (BrE and IndE), at least to some degree in spontaneous speech. More importantly, however, it was shown that a high degree of pitch variation in terms of range and dynamism is not as much characteristic of TrinE as a whole as it is of a specific group, namely (most) female Trinidadian speakers. It is these female Trinidadian speakers who differ from speakers of BrE and IndE in terms of their pitch variation patterns. We may conclude from these findings that Trinidadian speakers rely on endocentric norms at the prosodic level, but differently so across genders.

In fact, an important finding of this study is that pitch variation patterns are not homogenous in TrinE, but sociolinguistically conditioned in a systematic way, namely across gender, age, and ethnic groups, rural and urban speakers, or an interaction of different sociolinguistic variables. Some of this variation might be indirectly related to a notion of standard TrinE prosody which is centered on distance from the local Creole (see Deuber & Leung, 2013: 309). In essence, the findings reveal that there is a considerable degree of systematic local differentiation in TrinE prosody, as is common of later stages in the development of New Englishes, after endonormative stabilization has been achieved (Schneider, 2007: 52–55). On a more general level, the findings may be taken to indicate that endocentric tendencies—in the sense of the production of distinct, local prosodic patterns—and sociolinguistic differentiation in TrinE prosody are interlinked. Such an interpretation is in line with recent advances in World Englishes modeling, which assume that processes of endonormative stabilization and differentiation may frequently be merged (Buschfeld, 2020; Schröder & Zähres, 2020).

Our findings further indicate that, on a phonetic level, a high degree of variability in pitch could play a role in the popular perception of TrinE being ‘sing-song’ (see Youssef & James, 2008: 334), at least for female Trinidadian speakers. This phonetic pattern may contribute to previously identified intonational aspects on the phonological level, particularly accentual phrasing (Drayton, 2013; Ferreira & Drayton, 2017; Gooden & Drayton, 2017). Among female speakers (in spontaneous speech), a generally lower pitch level paired with a wider pitch range and a higher degree of pitch dynamism could potentially serve as phonetic cues for listeners and explain why Trinidadians speakers are often described as lilting (see Ferreira & Drayton, 2017: 24). In the absence of perceptual data, however, this remains a hypothesis to be verified in future speech perception research.

In view of the nature of the current study and its specific focus, future research should address the following issues. (1) Given that only a low number of rural and older speakers were represented in the sample (see Section 5), future studies are needed to verify the effects observed here. (2) Further research should address pitch range differences between TrinE and other Caribbean Englishes, and differences among the same Trinidadian speakers when speaking TrinE and TEC, respectively. A comparison of these aspects in both standard TrinE and TEC would allow for more specific and reliable conclusions concerning the influence of Creole on the local standard at the prosodic level. (3) It would also be worthwhile to include (standard) American English in such a cross-varietal comparison, considering growing American English influence in the Caribbean (e.g., Mair, 2009 on Jamaican English). (4) While the current study has analyzed data from one of the central domains of standard English usage in Trinidad, prosodic research based on data from other domains, such as media, business, or politics, possibly also including other speaking styles, would be worthwhile to investigate potential domain-specific differences (e.g., Deuber & Leung, 2013: 311). (5) Considering the scarcity of prosodic research on TrinE (as opposed to TEC) in general, research focusing on the intonational phonology of TrinE is also needed, and phonetically driven research should be extended to account for other potential measures of the global distribution of F0 (e.g., Barbosa, Madueira, & Mareüil, 2017). Once phonological research on the intonation of TrinE is available, an investigation of pitch range linked to specific tones (e.g., Mennen et al., 2012) would also be desirable. (6) Perceptual research would permit an evaluation of the degree to which the phonetic differences in pitch variation observed here are perceptually relevant.

## Notes

1. A preliminary analysis of part of the data included in this study was published as Meer and Fuchs (2019).

2. A notable exception is spoken English in the Bahamas, which is more influenced by American English due to the country’s long-lasting ties with the American mainland (Kraus, 2017). Little systematic research exists on accent variation in English in the smaller Caribbean countries and potential influences from British and American English outside the domain of radio newscasting (Hänsel & Deuber, 2019; Hänsel, 2021.).

3. Although downgraded in comparisons to Indo-Trinidadians, Afro-Trinidadian speakers still received relatively neutral overall ratings.

4. In addition, 18 of the 24 teachers produced short stimuli for an unrelated verbal guise test. These comprised no more than 1–2 (in a few cases up to 3) minutes of a total duration of about 30 minutes for the entire interview. The tasks used to elicit these short stimuli (picture description, giving directions on a map, giving study tips for students) also prompted speakers to produce declarative utterances. While these stimuli differ to some extent from the semi-structured, directed conversations in the interview, we submit that any potential task-based influences in our analyses are negligible, considering that (1) our analyses rely on global F0 parameters that solely take into consideration the overall distributional characteristics of F0 (see Section 5.2), (2) these parameters were further chosen to limit confounding effects of potential outliers in the F0 distribution (see Section 5.2), and (3) the overall duration of these stimuli is exceedingly short in comparison to the conversational component of the interview.

5. Apart from one female teacher (25 years old), the young age group solely consists of students. However, no differences were observed compared to the (female) students with regard to any of the F0 parameters in both speaking styles. The two older age groups only consist of teachers.

6. Note that this differs from the methods used in Meer and Fuchs (2019).

7. This categorization is based on the 2018 estimate for life expectancy in Trinidad and Tobago, which was 73.4 years (CIA, 2019), the 2011 median population age of 32.6 years, and the overall distribution of the population by age (Republic of Trinidad and Tobago, 2012: 1–9) .

8. Following guidelines for mixed-effects modeling (e.g., Twisk, 2006: 82), potential random slopes were fitted in a forward manner, that is, by adding by-school random slopes to a model with a random intercept for school and all fixed effects of interest (main effects and interactions; see Table 3).

9. Although it partially functions as a control variable in the current study, gender is not a random effect; the variable levels are not sampled from a larger population (e.g., Field, 2018: 943; Johnson, 2009: 365).

10. In most cases, both information criteria converged. In the small number of cases where AIC and BIC did not converge, we considered the former in the model building process because the latter tends to be overly conservative in non-large datasets (following Matuschek et al., 2017 308; see Vandekerckhove et al., 2015: 306).

11. While the interaction of variety and gender was overall significant, none of the pairwise comparisons reached significance. This might be due to the relatively conservative adjustment of the alpha level by the Bonferroni method in the case of a high number of pairwise comparisons. We therefore report *p*-values in Figure 2 (but not in the text) for all pairwise comparisons that involve TrinE and have a *p <* 1.

12. The reader should note that the older age group does not contain any Indo-Trinidadian speakers. The deviation metric calculated for this age group solely concerns the difference in pitch dynamism between Afro- and mixed-identifying Trinidadians.
